# A Recipe for Achieving Aichi: Conservation Planning for 2020 Biodiversity Targets

**DOI:** 10.1371/journal.pbio.1001892

**Published:** 2014-06-24

**Authors:** Jonathan Chase

**Affiliations:** Freelance Science Writer, Saint Louis, Missouri, United States of America

While much remains to be known, decades of careful research have documented rapid global declines of biodiversity at the hands of humans, perhaps approaching rates not seen since the last mass extinction more than 65 million years ago. Furthermore, in addition to well-founded moral and ethical reasons to be concerned about and mitigate biodiversity loss, recent years have seen a skyrocketing recognition by scientists, governmental policy-makers, and the general public of more self-interested values of biodiversity, including economic returns, mitigation of global changes, and benefits to human health.

**Figure 1:**
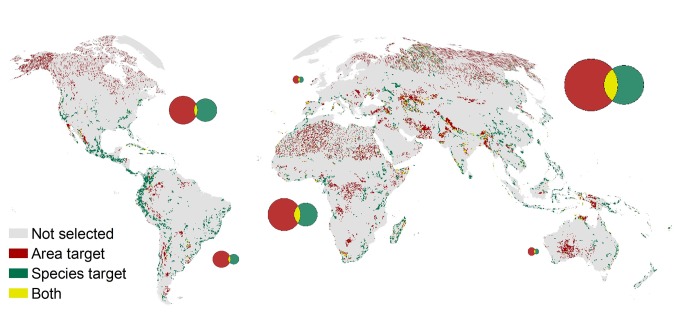
A mismatch of priorities. The map shows the distribution of priorities for establishing new protected areas to meet the 17% targets under Aichi Target 11. Red indicates protection at minimal cost and ignoring ecological representation. Green indicates protection that targets threatened species. Yellow indicates areas that are covered under both scenarios. *Image Credit: Dr. Oscar Venter, University of Queensland, Australia.* doi:10.1371/journal.pbio.1001891.

As a consequence of the recognition of the value of biodiversity and the need to quell its loss, several international consortia have devised various goals for the conservation of biodiversity. One of the more globally significant of these is the United Nations–initiated Convention on Biological Diversity, which in 2010 established a series of goals for the upcoming decade—collectively known as the Aichi Targets (after the location where the meeting took place: Aichi Prefecture, Japan). These targets include achieving greater awareness of biodiversity loss and the value of biodiversity, more sustainable use of resources for the protection of biodiversity, and safeguarding against future losses of biodiversity.

Because the primary driver of biodiversity loss is habitat loss, one of the main strategic goals of the Aichi Targets includes increasing the amount of protected terrestrial habitat (excluding Antarctica) from the current 13% to 17% across the globe by 2020 (Aichi Target 11). With nearly 200 nations agreeing to the principles of the Aichi Targets, this could lead to the most rapid rate of land preservation in history, even if the targets are not fully achieved. Another key goal is to prevent the extinction of species already known to be threatened with future extinction and to achieve improvement towards sustainability in their populations by 2020 (Aichi Target 12).

On the surface, achieving these two targets might seem quite complementary; preserving land should directly benefit the preservation of threatened species. Unfortunately, the ecological demons that have plagued scientists and policy-makers for decades by making what might seem to be a simple relationship on the surface into something much more complex are hard at work to thwart the Aichi targets. As pointed out by Venter and colleagues in this issue of *PLOS Biology*, the targets for protecting land and for protecting threatened species are not necessarily congruent, and in fact, a “business-as-usual” approach for land preservation to achieve the 17% target will do very little to increase the protection of threatened species. The crux of the argument boils down to the fact that not all land is equal when it comes to biodiversity preservation; there are biodiversity hotspots and biodiversity coldspots. And unfortunately, biodiversity hotspots, which tend to contain a higher proportion of threatened species, also have a tendency to have high economic value for uses other than preservation, such as agriculture.

To quantify current and future protection of threatened species within preserved areas, Venter and colleagues overlaid publically available data on the distribution of protected areas across the terrestrial extent of the globe (excluding Antarctica) and the ranges of several International Union for the Conservation of Nature (IUCN) Red List “threatened” species. Of the 4,118 threatened species they considered, 17% of them are not contained in a single protected area and only 15% (603) are considered to be adequately protected by habitats currently set aside for biodiversity. Importantly, these numbers are not fundamentally different from those calculated a decade ago, indicating disappointingly little progress.

Next, Venter and colleagues estimated the proportion of threatened species which could become protected through achieving 17% of land protected as mandated by Aichi Target 11. As a first pass, they assumed that governments would behave largely using a “business-as–usual” strategy for land preservation, meaning they would establish preserves in areas that have the least potential value for other uses (e.g., agriculture), allowing them to achieve the target area while minimizing the lost economic opportunity costs. These less economically valuable habitats, however, also tend to be less productive or otherwise constrained in the numbers of threatened species they house. As a result, Venter and colleagues predict that despite achieving the 17% land preservation of Aichi Target 11, only 249 more threatened vertebrate species would be adequately protected with this extended network, leaving 79% of these threatened species still at relatively high risk and doing little to achieve Aichi Target 12 (i.e., sustainability of threatened species).

Notably, the Aichi Target 11 has wording designed to encourage land preservation strategies beyond a simple area target, including phrases for preservation such as “especially areas of particular importance for biodiversity and ecosystem services” and “ecologically representative.” To examine the potential of the latter criterion, Venter and colleagues examined a scenario where future land preservation was equally distributed among ecoregions of different vegetative communities. Again, they find only a marginal benefit in terms of numbers of threatened vertebrates which gained adequate protection, despite a 450% increase in lost-opportunity cost of setting land aside, relative to the “business-as–usual” scenario. They estimated the potential lost-opportunity costs of setting aside land that would ensure the adequate protection of all 4,118 threatened species to be nearly $43 billion (in US dollars), around 750% more opportunity cost lost than with “business-as–usual.”

How then, can we reconcile the low economic cost but low conservation benefit scenario of “business-as–usual” designation of protected areas, given the exceedingly high economic costs that it would take to set aside land to achieve Aichi Target 11's 17% land protection goals—along with Target 12's mandate to minimize extinctions of already threat ened species? Importantly, Venter and colleagues identified a critical nonlinearity in the relationship between the costs of establishing new reserves and the benefits of achieving adequate protection of threatened species. That is, small increments of higher lost-opportunity cost lead to proportionately larger increments of adequate protection of threatened species. For instance, achieving a 400% increase in the adequate protection of threatened species only costs 50% more, in terms of lost-opportunity cost, than the “business-as-usual” strategy.

There are, of course, many caveats inherent in the specific estimates put in place by Venter and colleagues. However, the nonlinearity is likely a robust result that points towards a “happy medium” where countries can gain considerable benefits in terms of biodiversity preservation with minimal lost-opportunity costs by incorporating considerations of threatened species into their networks of protected areas. Hopefully, the Aichi Targets will enjoy better success than many previous goals from the Convention on Biodiversity Conservation that were ultimately left unrealized, perhaps because the goals were unrealistic and/or because they were not well placed within socioeconomic constraints. By providing a better road map towards achieving biodiversity conservation goals within an explicit socioeconomic framework, the analysis by Venter and colleagues provides a step towards accomplishing these targets. And none too soon—the front page of the Convention on Biodiversity's website (http://www.cbd.int/) indicates that there are less than 2,500 days to achieve the Aichi Targets…and counting.

**Venter O, Fuller RA, Segan DB, Carwardine J, Brooks T, et al. (2014) Targeting Global Protected Area Expansion for Imperiled Biodiversity.** doi:10.1371/journal.pbio.1001891

